# Effect of environmental factors on hematology profile of *Gambusia affinis* caught at Brantas River watershed, Indonesia

**DOI:** 10.12688/f1000research.74117.1

**Published:** 2021-11-17

**Authors:** Asus Maizar Suryanto Hertika, Diana Arfiati, Evellin Dewi Lusiana, Renanda B.D.S. Putra

**Affiliations:** 1 Water Resource Management, Universitas Brawijaya, Malang, East Java, 65144, Indonesia; 2 Aquacultue PSDKU, universitas Brawijaya, Kediri, East Java, 64111, Indonesia

**Keywords:** biomarker, blood parameter, ecosystem health, water quality, river pollution

## Abstract

**Background**: Contamination of freshwater ecosystems has become a major issue as it threatens public water sources as well as aquatic life. It is important to predict changes in organism health, given a known number of environmental factors and pollutant concentrations, in order to better manage contaminants through biomarker analysis. This study aims to examine the ecosystem health of the Brantas River based on its environmental condition and the hematology profile of *Gambusia affinis* fish present in the river. This species was chosen because of its wide distribution along the Brantas River, and because it is very tolerant, adaptable, highly abundant, and easy to catch.

**Methods**: The study area included 10 sampling sites along the Brantas River watershed. In total, six water quality parameters were observed (temperature, pH, dissolved oxygen (DO), biological oxygen demand (BOD), ammonia concentration, and phenol concentration) and hematology measurements consisted of erythrocyte, leucocyte, and micronuclei analyses.

**Results**: The results showed that the upstream area of Brantas River, located in Batu, was the least polluted region, while Mojokerto was the most polluted. The erythrocyte level of *Gambusia affinis* caught in most sampling sites was quite low. Furthermore, research revealed that the status of *Gambusia affinis*' hematological profile was significantly correlated (p<0.05) with water quality parameters, particularly DO, BOD, ammonia, and phenol.

**Conclusions**: It can be concluded from these results that the hematological profile of the fish is poor due to high levels of organic waste and harmful substances.

## Introduction

The contamination of freshwater ecosystems with a variety of pollutants has become a major concern in recent decades. ^1^^,^^2^ This is not only because of hazards to public water sources, but also because of the harm done to aquatic life. ^3^ Pollution from both point and non-point sources can have an impact on water quality in freshwater ecosystems. ^4^ Contamination from a single identifiable source, such as industrial effluents and wastewater treatment plants, is known as point source pollution. ^5^ Non-point sources include runoff linked to certain land use patterns, for example storm water and sewage outflows in urban areas, or fertilisers, pesticides and animal manure in agricultural or forested areas. ^6^ Changes in abiotic elements such as precipitation and temperature levels resulting from climate change can also impact the regular functions of aquatic ecosystems, including reproduction and feeding. ^7^

Environmental contaminants can impair aquatic organisms' survival in a range of ways, including direct toxicity (both short- and long-term). ^8^ Usually, the effects are insidious, eventually affecting organism health. ^9^ Common laboratory metrics of growth, reproduction, and survival can be poor predictors of the myriad indirect stressor effects which exist in the field. ^10^ It is therefore necessary, given a known number of environmental factors and pollutant concentrations, to be able to forecast changes in ecosystem structure and function, as well as organism health in order to better manage contaminants. ^8^ Biomarker analysis of organisms gathered in the field is appropriate for this purpose because it can offer information on the state of the environment without the requirement for extrapolating laboratory results, which is associated with uncertainty. ^11^

The use of particular biomarkers for assessing environmental quality and the health of fish in degraded environments has proven to be useful and widely-used. ^12^^,^^13^ Fish are commonly employed to assess the quality of the aquatic environment and are recognized as bio-indicators of pollution. ^14^^,^^15^ Fish live in close proximity to their environment and are thus vulnerable to physical and chemical alterations to it, which may manifest in components of their blood. ^16^ Hematological characteristics are an excellent diagnostic for physiological dysfunction, since their values exhibit genetic and physiological variations. ^17^^–^^19^ Genetic variation can be caused by both interspecific and intraspecific influences within species. ^20^ Moreover, differences between hematological values can be used to assess the links between these variables, and correlate them with the state of an organism's health in response to environmental conditions. ^21^^,^^22^ The species utilized in biomonitoring programs must be sensitive, and the biomarkers evaluated should provide a consistent response. ^23^

The Brantas River, located in the East Java province of Indonesia, is the second largest river on Java Island. ^24^ It is prone to various types of contamination because of the extensive human activities along the course of the river: Java Island is the most populated region and largest industrial center in Indonesia. ^25^ The Brantas River is rich in aquatic biota, including *Gambusia affinis* which has frequently been used in biomonitoring activities in many previous studies. ^11^ This species was used in this study because it has a wide distribution along the course of the Brantas River, ^26^ and is also very tolerant, adaptable, abundant, and easy to catch. ^11^^,^^27^ Assessment of water quality in the Brantas River using common methods such as pollution indices and the Storet method have been performed in several preceding studies. ^25^^,^^28^^,^^29^ However, understanding of the ecosystem health of Brantas River, which is associated with the biological systems of aquatic biota, is still limited. Therefore, the purpose of this study is to examine the ecosystem health of the Brantas River based on its environmental condition and the hematological profile of *Gambusia affinis* present in the river.

## Methods

### Ethical considerations

Ethical approval was not needed for this research as the fish species used is highly abundant in the research area and not classified as an endangered species according to International Union for Conservation of Nature (IUCN) list. Moreover, according to the Regulation of the Indonesia Minister of Marine Affairs and Fisheries Number PER.17/MEN/2009 the fish species is not protected.

### Study area

**Figure 1.**
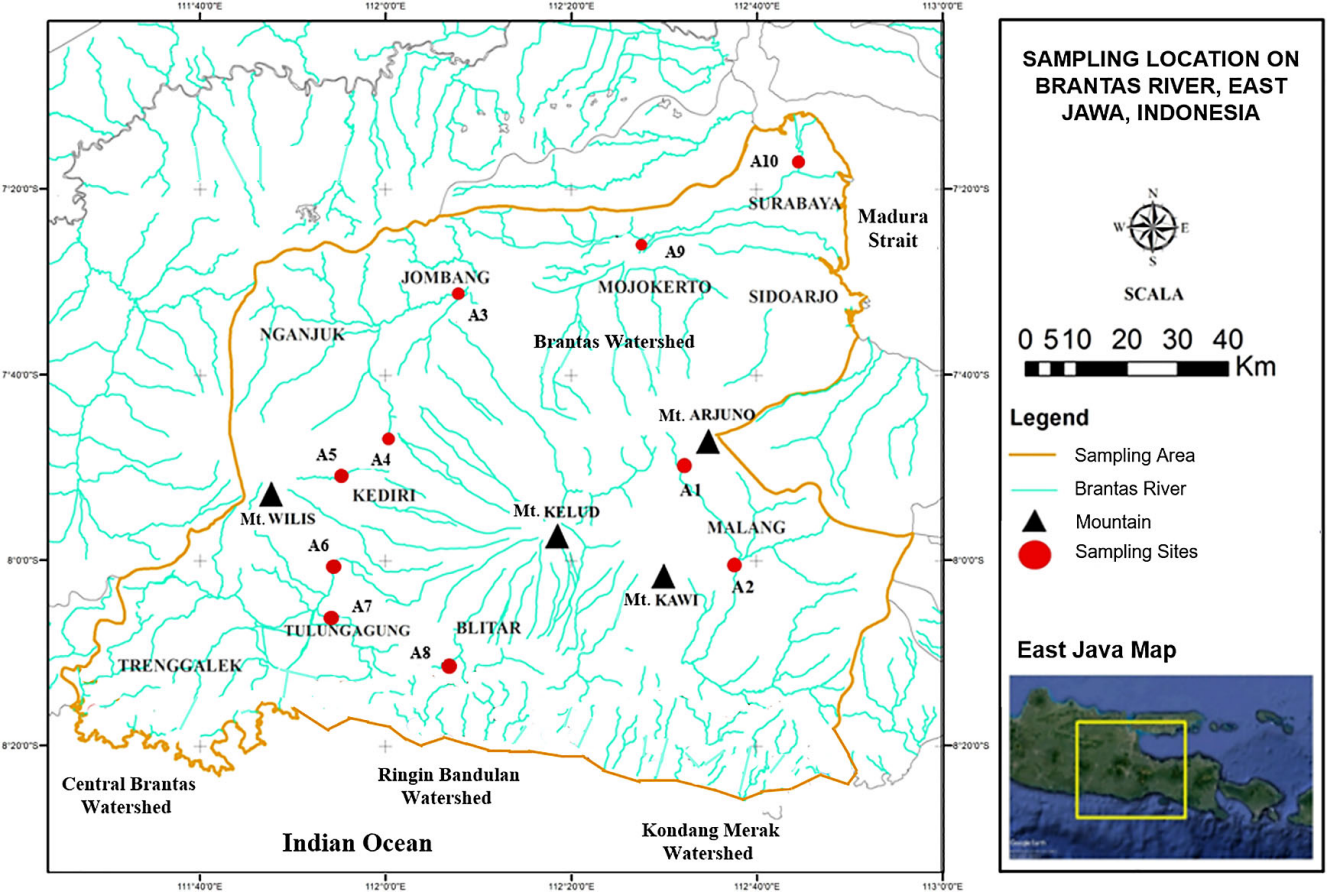
Sampling locations in the Brantas River watershed.

This research was carried out in the Brantas River. In total, ten sampling sites were chosen based on the geomorphological characteristics of the Brantas River watershed, as depicted in Figure 1. Each sampling site was divided into three carefully chosen sub-sites. Sampling was performed monthly between February and April of 2019.

### Water quality measurement

Several physico-chemical water quality parameters were measured in this research in order to assess the environmental condition of the study area. The parameters were temperature, pH, dissolved oxygen (DO), biological oxygen demand (BOD), ammonia (NH _3_) concentration, and phenol concentration. The methods and instruments utilized for these measurements are presented in Table 1.

**Table 1.** Methods and instruments used for water quality evaluation.

Parameter	Unit	Method/Instrument
Temperature	°C	Lutron PDO-520
pH	-	pH meter
Dissolved Oxygen (DO)	mg/L	Lutron PDO-520
Biological Oxygen Demand (BOD)	mg/L	Winkler method ^30^
Ammonia (NH _3_)	mg/L	Spectrophotometer Genesys 10S UV-Vis
Phenol	mg/L	4-Aminoantipyrine method ^32^

For BOD calculation, we measured the initial dissolved oxygen level at the beginning of sampling period, then re-measured it after incubation with a temperature of 20 ^o^C for 5 days by using the Winkler method. ^30^^,^^31^ In the Winkler method or iodometric technique, divalent manganese solution is added to the solution in a glass-stopper bottle, followed by the addition of strong alkali. DO quickly oxidizes an equal quantity of distributed divalent manganese hydroxide precipitates to higher valence state hydroxides. When oxidized manganese is exposed to iodide ions in an acidic solution, it reverts to the divalent state, releasing the iodine equivalent of the initial DO concentration. The iodine is then titrated with a stranded thiosulfate solution. A starch indicator can be used to visually determine the titration end point. ^30^ The BOD value was the difference between the initial and final dissolved oxygen level. Moreover, phenol level was estimated using 4-aminoantipyrine method. ^32^ The phenolic compound was mixed with 4-aminoantipyrine along with alkaline oxidant at high solution pH, which formed a red quinone dye. ^33^

### Fish blood sample collection and hematology study

We took mosquito fish ( *Gambusia affinis*) directly from the Brantas River from ten sampling sites. The fish were caught using nets on the banks of the river. The fish samples were in good health. The fish species includes the endemic fish of the Brantas River which are cosmopolitan (available at every station) and in large numbers. Each sampling site consisted of 3 sub-sites, and we took 3 fish at every sub-sites. Hence, we used a total of 90 fish in this study. The fish we took were adult fish with sizes ranging from 5 to 6 cm with a weight of 4-5 grams. The samples taken were female adult fish which have a larger body size than male fish. Therefore, the female adult fish had sufficient blood volume for hematology analysis.

Blood samples of *Gambusia affinis* were collected straight from the lateral line of the caudal fin. These samples consisted of roughly 90 μL of blood collected using a 1 mL syringe with 10 μL sodium citrate anticoagulant. This method of extracting blood from the fish was lethal. The samples were moved to a 1.5 mL Eppendorf flask and homogenized. ^34^ The homogenization was done by shaking the Eppendorf flask manually. After that, the samples were placed in a cool box with ice gels and promptly delivered to the Laboratory of Parasites and Fish Diseases, Faculty of Fisheries and Marine Sciences, Brawijaya University, Malang, Indonesia and immediately observed.

The hematological parameters measured in this study were the total values of erythrocytes, leucocytes, and micronuclei. A hemocytometer was used to quantify erythrocytes and leucocytes. ^35^^,^^36^ Micronuclei were measured in accordance with micronuclei test procedures. ^37^

### Data analysis

Data produced in this study were statistically analyzed, both descriptively and inferentially. For descriptive analysis, numerical measures (mean and standard deviation) and graphical technique (boxplot and scatter plot) were employed. For inferential analysis, one-way analysis of variance (ANOVA) and the Tukey test ^38^ were utilized to compare variables across sites, and Pearson correlation analysis ^39^ was used to analyze relationships between environmental conditions and hematological characteristics. For ANOVA, we also conducted assumption test such as normality (Shapiro-Wilk test) and homoscedasticity (Bartlett test). All of these analyses were performed using the statistical analysis software R (version 3.6.1).

## Results and discussion

### Water quality factors in Brantas River

Table 2 shows that the Batu and Malang sites had the lowest water temperatures, while water temperature in the other regions was higher (90). Batu is located at a high elevation in the upstream area of the Brantas River. ^28^^,^^40^ Malang is situated next to Batu, but has a high population density related to intensive residential development and industrial and agricultural activities. ^41^ Acidity levels at all sampling sites were considered normal, since the pH values recorded all lay within the recommended range of 6.5-8.5. ^42^ Fluctuations in pH values are influenced by the discharge of organic and inorganic waste into river bodies. ^43^ Very low pH conditions can result in the death of aquatic biota. ^44^

**Table 2.** Physicochemical water quality factor in Brantas River.

Site	Temperature (°C)	pH	DO (mg/L)	BOD (mg/L)	Ammonia (mg/L)	Phenol (mg/L)
Batu	23.52 ± 0.34 ^g^	7.48 ± 0.08 ^ab^	7.91 ± 0.25 ^a^	8.17 ± 0.90 ^g^	0.45 ± 0.07 ^f^	0.65 ± 0.05 ^c^
Malang	27.10 ± 1.01 ^f^	7.38 ± 0.10 ^abc^	6.46 ± 0.27 ^cd^	10.34 ± 0.60 ^f^	0.61 ± 0.04 ^e^	0.77 ± 0.04 ^bc^
Jombang	29.37 ± 1.10 ^abc^	7.29 ± 0.07 ^bcd^	6.38 ± 0.05 ^cd^	11.94 ± 0.74 ^e^	0.66 ± 0.04 ^de^	0.71 ± 0.06 ^bc^
Kediri1	28.44 ± 0.59 ^cde^	6.87 ± 0.15 ^f^	6.59 ± 0.22 ^c^	12.41 ± 0.96 ^de^	0.73 ± 0.03 ^bcd^	0.80 ± 0.04 ^b^
Kediri 2	30.22 ± 0.13 ^a^	7.42 ± 0.10 ^abc^	7.46 ± 0.07 ^b^	13.33 ± 0.09 ^cd^	0.78 ± 0.04 ^bc^	0.77 ± 0.05 ^bc^
Tulungagung 1	30.16 ± 0.34 ^ab^	7.49 ± 0.05 ^a^	7.32 ± 0.07 ^b^	13.96 ± 0.32 ^bc^	0.79 ± 0.03 ^b^	0.76 ± 0.04 ^bc^
Tulungagung 2	27.87 ± 0.15 ^ef^	7.26 ± 0.16 ^cd^	6.50 ± 0.34 ^cd^	14.22 ± 0.32 ^bc^	0.68 ± 0.07 ^cde^	0.72 ± 0.07 ^bc^
Blitar	28.19 ± 0.31 ^def^	7.42 ± 0.13 ^abc^	7.10 ± 0.29 ^b^	14.23 ± 0.13 ^bc^	0.49 ± 0.03 ^f^	0.70 ± 0.04 ^bc^
Mojokerto	29.08 ± 0.74 ^bcd^	7.17 ± 0.10 ^de^	6.12 ± 0.28 ^d^	18.40 ± 0.85 ^a^	1.11 ± 0.20 ^a^	1.14 ± 0.24 ^a^
Surabaya	29.73 ± 0.29 ^ab^	6.98 ± 0.12 ^ef^	6.68 ± 0.41 ^c^	14.77 ± 0.27 ^b^	0.66 ± 0.05 ^de^	0.77 ± 0.04 ^bc^

DO values measured in Batu, Kediri, and Blitar were higher than those measured in other regions. The lowest DO value was found in Mojokerto. Nevertheless, the results of DO measurement were all above 6 mg/L. The optimum DO for fish is generally greater than 6 mg/L. ^45^ If DO levels are not favourable, this will cause stress to fish because the brain will not be supplied with enough oxygen. This condition can cause death due to lack of oxygen (anoxia), as the fish body tissue will not be able to bind oxygen dissolved in the blood. ^46^ BOD measurement results contrasted with DO results. The lowest BOD level was found in Batu, and the highest in Mojokerto. Some BOD values (Tulungagung, Blitar, Mojokerto, Surabaya) were beyond the regulatory standard level (14 mg/L) set by the Indonesian Ministry of Environment. ^47^ An increase in BOD can disrupt biota, because it is accompanied by a decrease in DO. ^48^ This happens because of the disruption of the activity of bacteria that break down waste and foreign substances in the water. ^49^ Thus, the higher the BOD, the more polluted the waters. ^48^

Ammonia and phenol levels in the Brantas River watershed ranged from 0.45 to 1.11 mg/L and 0.65 to 1.14, respectively. The variation in concentration of these parameters was similar to that of BOD, in that Mojokerto had the highest value. According to the Indonesian Ministry of Environment, the maximum permittable value of ammonia is 0.5 mg/L and that of phenol is 0.001 mg/L. Therefore, the majority of ammonia measurements in the study area surpassed the standard, while all of the phenol values measured in this study were far beyond the standard. Ammonia can diffuse through fish body tissues if the concentration is high, and it can potentially be toxic. ^50^ The level of ammonia toxicity is influenced by several factors, including fish species, level of exposure to ammonia, duration of exposure, and the effect of acclimatization discussed previously. ^51^ Fish exposed to high levels of ammonia will experience damage to their gills and blood cells. ^52^ Erythrocyte levels will decrease as ammonia levels in the water increase. ^53^ Sources of phenol include waste discharged from various industries involving the production and disposal of coal, phenols, pharmaceuticals, resins, paints, textiles, leather, and petrochemicals. ^54^ Exposure of animals to phenol through inhalation causes liver damage, kidney damage, neurological effects, developmental effects, skin effects and even death. ^55^^,^^56^ The presence of phenol in excess of the water threshold can be a chemical stressor for aquatic organisms. ^57^

The physico-chemical conditions of the Brantas River watershed indicate that the Batu region was the least polluted site, since the values of its water quality factors were better than the other sites. On the other hand, Mojokerto is the most polluted region, as half of its water quality parameters surpassed the standard values. One-way ANOVA and Tukey test results also suggested that the environmental states of the sampling sites, specifically Batu and Mojokerto, were significantly different, as none of the water variables in these regions shared the same letter notation.

### Hematology profile of *Gambusia affinis* caught in the Brantas River

**Figure 2.**
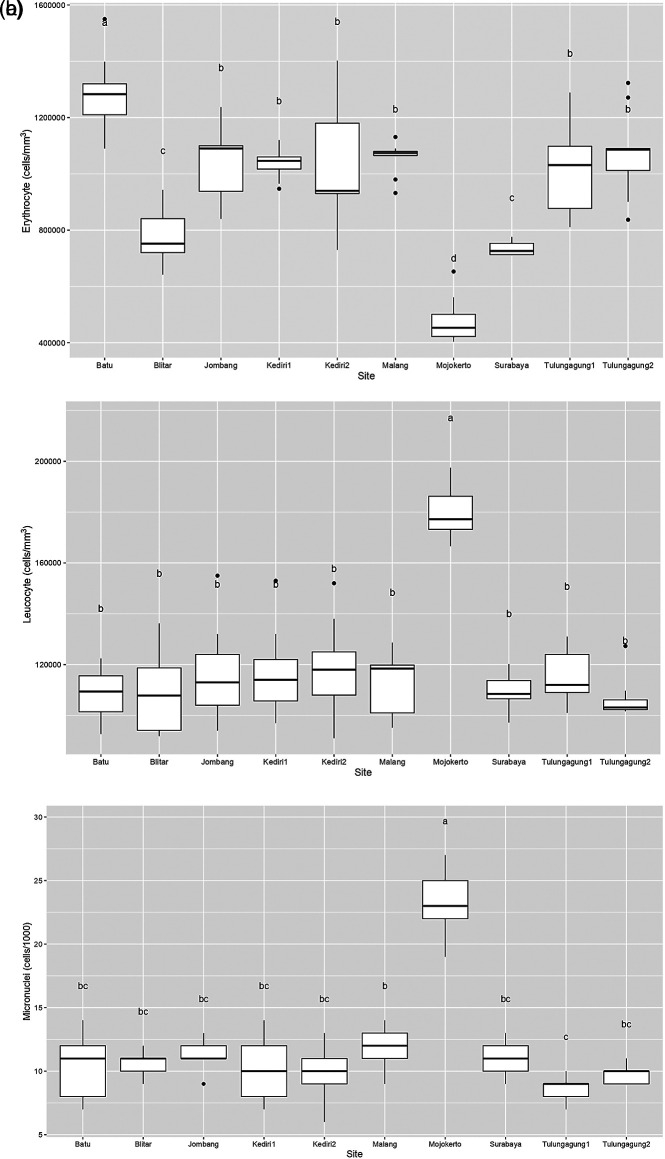
Boxplot of *Gambusia affinis*’ hematology profile which caught in study area (a) Erythrocyte; (b) Leucocyte; (c) Micronuclei.

The hematology profile of fish is one of the early warning parameters that can be used to assess the level of fish health in relation to the aquatic environment. ^18^ If the environment is favorable, the blood parameters will also show normal conditions. Otherwise, the hematology profile of fish will show abnormal conditions when the environment is polluted. ^58^ Figure 2(a) is a box plot of the erythrocyte count of *Gambusia affinis* samples caught at each of the sampling sites. It shows that the highest erythrocyte level was found in Batu, with a median of around 1,300,000 cells/mm ^3^. Meanwhile, the erythrocyte levels of *Gambusia affinis* samples from Jombang, Kediri, Malang, and Tulungagung were recorded as slightly more than 1,000,000 cells/mm ^3^. Samples from both Blitar and Surabaya had erythrocyte levels slightly below 800,000 cells/mm ^3^. Lastly, the erythrocyte levels in samples obtained in Mojokerto were measured as around 400,000 cells/mm ^3^.

It is clearly visible in Figure 2(b) that a large number of sampling sites resulted in leucocyte values which ranged between 1,000,000 and 1,400,000 cells/mm ^3^. Leucocyte levels in *Gambusia affinis* caught in Mojokerto were significantly higher, at more than 1,800,000 cells/mm ^3^. Similarly, micronuclei levels in fish from this sampling site were around 25 cells/1000: this was the highest level found in this study ( Figure 2(c)). Recorded micronuclei levels in *Gambusia affinis* from the remaining sites were a little above 10 cells/1000.

Erythrocytes are blood components which play a role in the process of transporting O _2_ and CO _2_ for respiration and in nutrient metabolism in fish. ^59^ When oxygen and iron levels in the cytoplasm increase, reactive oxygen in the form of O _2_^−^ (superoxideanion), H _2_O _2_ (hydrogen perioxide) and OH (hydroxyl radical) are produced. ^60^ Erythrocytes are very sensitive to the oxidative processes of unsaturated fatty acids present in the membrane. ^61^ In fish and other aquatic animals, erythrocytes can be damaged by pollutants dissolved in the water. ^62^

Leukocytes play an important part in the immune system. These cells help rid the body of foreign substances, including invading pathogens, through the immune response system. ^63^ Unhealthy fish will produce high levels of leukocytes in order to phagocytize bacteria and synthesize antibodies. ^64^ As fish try to defend themselves from poor environmental conditions by producing antibodies due to exposure to bacteria, leukocyte levels increase. ^65^

Micronuclei are small extranuclear chromatins surrounded by a nuclear envelope containing DNA. ^66^ Micronuclei are formed during cell division at the anaphase stage when a chromosome fragment or part of a chromosome is not incorporated into a nucleus. ^67^ Micronucleus analysis performed on fish can be used to detect the clastogenic and aneugenic effects of genotoxic materials present in the waters. ^68^ Micronuclei analysis using blood cells is preferred. This is because blood cells are very sensitive, and because the kidneys, which are the main hematopoietic organs in fish, are easy to sample. ^69^ Micronuclei analysis is a method used to monitor aquatic pollutants with mutagenic properties. Fish can respond to mutagens present in aquatic habitats even at very low concentrations. These mutagens can lead to the formation of micronuclei in cells, with high levels of micronuclei formed in fish living in polluted waters ^70^

In normal fish, the number of erythrocyte cells ranges from 1,050,000 to 3,000,000 cells/mm ^3^, ^13^ while the number of leucocyte cells ranges from 20,000-150,000 cells/mm ^3^. ^71^ Therefore, erythrocyte levels in *Gambusia affinis* caught in Blitar, Surabaya, and Mojokerto can be considered low. On the other hand, all sampling sites indicate an extremely high number of leucocytes in the *Gambusia affinis* samples. This may suggest the presence of health problems in fish due to pollution. ^72^ According to the field survey and, ^26^ these regions are dominated by industrial, agricultural and dense residential areas where domestic wastes from various anthropogenic activities are channeled into river bodies, resulting in low erythrocyte counts. Physical or chemical environmental changes will affect the blood components of fish. Exposure of fish to certain chemical compounds can reduce erythrocyte levels. ^52^ Furthermore, the fish sampled at these sites experienced health problems such as gill damage, and injuries around the body and tail. Decrease in erythrocyte levels can be caused by deterioration of the gills disrupting their function, which has an impact on the ability of hemoglobin (Hb) to bind oxygen. ^73^ Reduced erythrocyte levels are also thought to be caused by anemia in fish. Anemia is a disorder which occurs in the blood tissue of fish. This disorder occurs as a result of the exposure of fish to chemical pollutants or heavy metals, resulting in dysfunction of the osmoregulatory organ. The supply of food to cells, tissues and organs will also be reduced, so that the metabolic processes of fish are hampered. ^74^^,^^75^ In addition, leukocyte and erythrocyte counts in fish are negatively correlated: the higher the number of erythrocytes, the lower the number of leukocytes. ^76^ Leukocytes help rid the body of foreign substances, including invading pathogens, through the immune response system. Unhealthy fish will produce more leukocytes in order to phagocytize bacteria and synthesize antibodies. ^64^

### Relationship analysis of hematology properties and water quality

**Figure 3.**
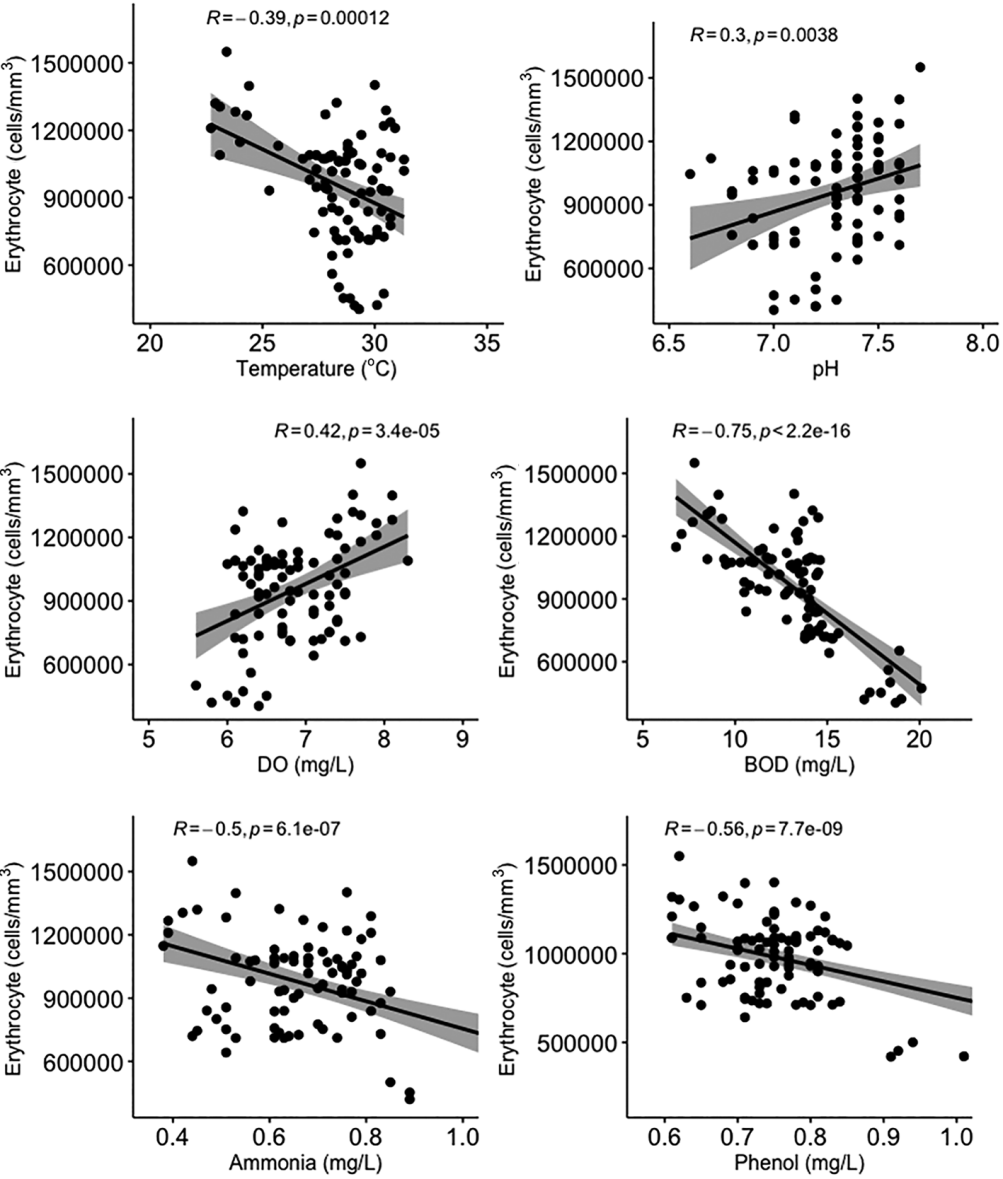
Scatter plot and Pearson correlation analysis between erythrocyte and water quality parameters. DO = dissolved oxygen; BOD = biological oxygen demand.

**Figure 4.**
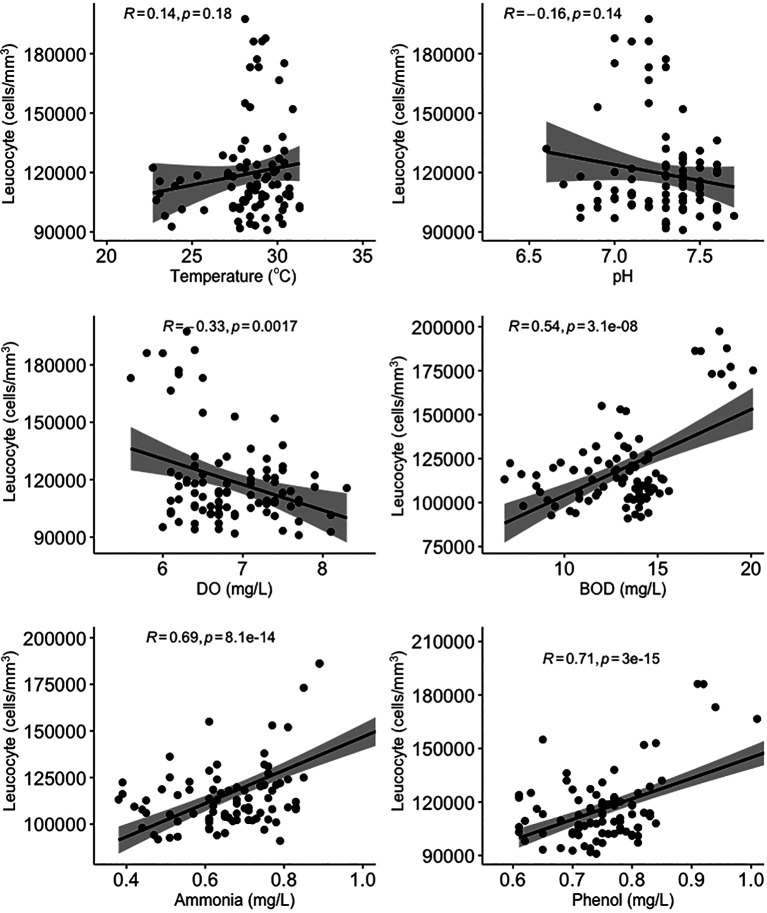
Scatter plot and Pearson correlation analysis between leucocyte and water quality parameters. DO = dissolved oxygen; BOD = biological oxygen demand.

**Figure 5.**
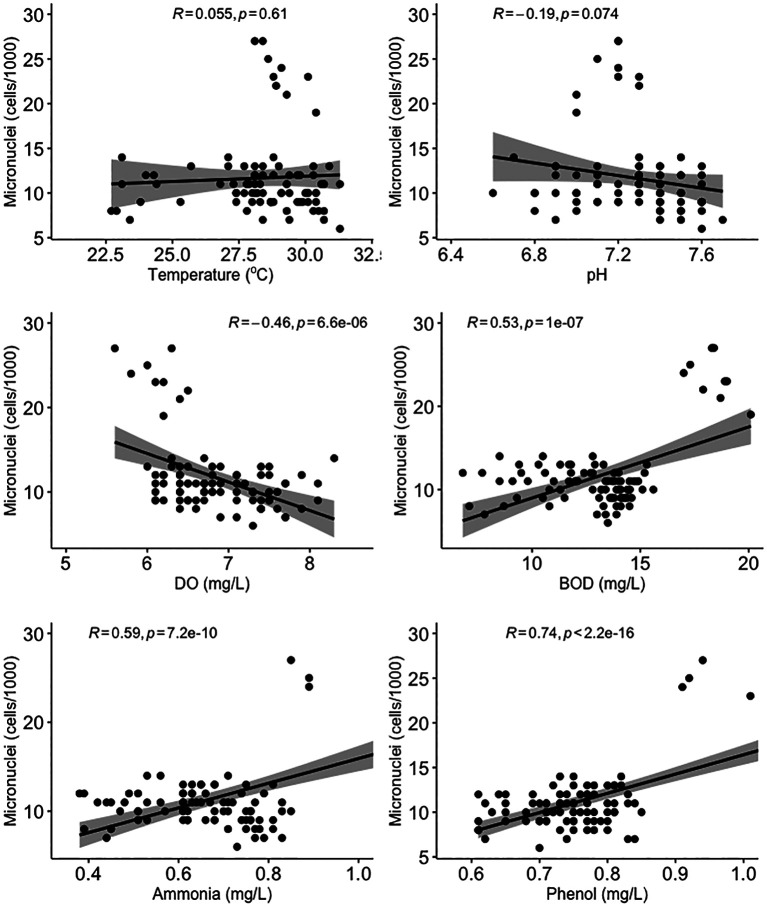
Scatter plot and Pearson correlation analysis between micronuclei and water quality parameters. DO = dissolved oxygen; BOD = biological oxygen demand.

All water quality parameters in this study were significantly correlated ( *p* < 0.05) with erythrocyte levels in *Gambusia affinis* caught in the Brantas River watershed ( Figure 3). Temperature, BOD, and ammonia and phenol concentrations were negatively correlated with erythrocyte levels, while pH and DO were positively correlated with erythrocyte levels. On the other hand, temperature and pH were not significantly correlated ( *p* > 0.05) with leucocyte levels ( Figure 4). DO was negatively correlated with leucocyte levels while ammonia and phenol concentrations along with BOD were positively correlated with leucocyte levels ( *p* < 0.05). As shown in Figure 5, there was no significant correlation between micronuclei and water quality factors.

Fish life is closely related to their habitat. Physical or chemical environmental changes will affect the blood components of fish. ^77^ Erythrocytes are very sensitive to the oxidative processes of unsaturated fatty acids present in the membrane. ^61^ Fish in poor health and fish in polluted environments have lower erythrocyte levels than healthy fish. ^78^ Stressed fish have increased leukocyte levels, as a reaction to variations in environmental circumstances or the presence of foreign substances. ^19^ However, the hematology profile of fish is not only caused by the environment but also by disease. Therefore, when taking fish blood, it is ensured that the sampled fish are in good health by showing normal morphological conditions and actively swimming in the water column.

Ammonia is the end product of organic and inorganic nitrogen metabolism. It can be toxic, and this can affect biota living in water. ^79^ Ammonia has a sub-lethal effect on fish, causing narrowing of the gill surface which results in a decrease in the speed of the gas exchange process in the gills. ^80^ Sub-lethal effects of ammonia can also include a decrease in the number of blood cells, a decrease in oxygen levels in the blood, reduced physical resistance and resistance to disease, and structural damage to various types of organs, including the liver parenchyma. ^81^ As a result, high levels of ammonia cause an increase in leucocyte levels and a decrease in erythrocyte levels. ^53^

BOD is commonly used to determine the level of water pollution and to trace the flow of pollution from upstream to estuary. ^82^ BOD is closely related to DO. As pollution increases in a body of water, BOD increases while DO decreases. ^48^ High BOD can cause problems in aquatic animals, for example paralysis in fish, because sufficient oxygen cannot be supplied to the brain. ^83^ This condition can also result in the death of fish due to lack of oxygen (anoxia), which is caused by the inability of fish body tissues to bind oxygen dissolved in the blood, which causes damage to blood cells. ^84^ High BOD can also affect micronuclei levels in fish. ^85^ This implies the existence of a toxic impact in which fish have been directly exposed to wastewater containing toxic substances where aneugenic and/or clastogenic activities are taking place. High micronuclei levels may indicate aneugenic and/or clastogenic activity associated with micronuclei formation due to poor spindle function of the chromosomes. Increases in micronuclei counts in fish can indicate an increase in wastewater levels in the river and this can cause environmental damage resulting in cytogenic damage to fish erythrocytes. ^86^

Phenol has high toxicity in water and can cause damage to both aquatic ecosystems and human health. Phenol can damage blood cells, the central nervous system and organs such as the kidney and liver, cause low blood pressure, and potentially be lethal. ^55^ A decrease in the number of erythrocytes can indicate the occurrence of anemia, which can be triggered by the entry of phenol into blood cells. ^87^ Phenol compounds have pathological effects which include gill necrosis, increased production of gill mucus, histological changes in the heart, liver, spleen and skin, and destruction of erythrocytes. This condition can also increase white blood cells in fish, which indicates poor health. In addition, high levels of phenol in the water can cause damage to the endocrine system, disrupt liver performance and have an impact on genotoxicity. ^88^ Genotoxic agents in fish are related to the frequency of pollution in the waters. Genotoxicity testing of the aquatic environment can be carried out using the micronuclei test. Micronuclei are formed from chromosomal fragments or entire chromosomes that are left behind during cell division due to lack of centromeres, defects in the centromere or defects during cytokinesis. In tissue division, the micronuclei test can determine the presence of clastogenic or aneugenic compounds. ^89^

## Conclusion

The Brantas River is one of the longest rivers on Java Island and is highly susceptible to pollution, especially as a result of anthropogenic activities. This study aimed to evaluate the ecosystem health of the Brantas River by linking physicochemical conditions and the hematological profile of *Gambusia affinis* caught in the river. The results indicate that the upstream area of the Brantas River located in Batu is the least polluted stretch of the river and is in good condition in terms of water quality, while all other areas of the river were in poor condition, especially in Mojokerto. With regard to the hematological profile of the sampled fish species, the erythrocyte levels of *Gambusia affinis* caught in most of the sample sites were quite low, while the leucocyte and micronuclei levels were high. This indicates the presence of contaminants in the water which have triggered the immune systems of the fish. Further investigation showed that the hematological profile of the *Gambusia affinis* samples significantly correlated with the water quality parameters measured, especially DO, BOD, ammonia, and phenol. It can therefore be concluded that the unsatisfactory hematology profile of the fish may primarily be due to the presence of high levels of organic waste and toxic substances in the river.

## Data availability

Figshare: Water quality parameters and hematology profile of Gambusia affinis caught at Brantas River watershed, Indonesia. https://doi.org/10.6084/m9.figshare.16895227.v1. ^90^

Data are available under the terms of the Creative Commons Zero “No rights reserved” data waiver (CC0 1.0 Public domain dedication).
