# Supplementary material for: Effect of environmental factors on blood counts of Gambusia affinis caught at Brantas River watershed, Indonesia
Source: F1000Res. 2022 Mar 23;10:1169. Originally published 2021 Nov 17. [Version 2] doi: 10.12688/f1000research.74117.2 (PMC8978147; doi:10.12688/f1000research.74117.2)
Supplement: Peer review history

# Peer review: Reviewer response for version 2

**DOI:** 10.5256/f1000research.122571.r128615

**Published:** 2022-03-30

**Authors:** Akhmad Mukti

**Affiliations:** 1 Department of Aquaculture, Faculty of Fisheries and Marine, Universitas Airlangga, East Java, Indonesia

I have reviewed and approved the revisions that have been made by the author in the manuscript

Is the work clearly and accurately presented and does it cite the current literature?

Yes

If applicable, is the statistical analysis and its interpretation appropriate?

Yes

Are all the source data underlying the results available to ensure full reproducibility?

Yes

Is the study design appropriate and is the work technically sound?

Yes

Are the conclusions drawn adequately supported by the results?

Yes

Are sufficient details of methods and analysis provided to allow replication by others?

Partly

Reviewer Expertise:

Fish genetics, fish reproduction, an endocrine disorder, genotoxic, karyology, aquaculture biotechnology

I confirm that I have read this submission and believe that I have an appropriate level of expertise to confirm that it is of an acceptable scientific standard.

# Peer review: Reviewer response for version 2

**DOI:** 10.5256/f1000research.122571.r128616

**Published:** 2022-03-30

**Authors:** Mohammad Tamrin Mohamad Lal

**Affiliations:** 1 Borneo Marine Research Institute, Universiti Malaysia Sabah, Kota Kinabalu, Malaysia

The manuscript was well revised. I have no further comment on this manuscript.

Is the work clearly and accurately presented and does it cite the current literature?

Partly

If applicable, is the statistical analysis and its interpretation appropriate?

I cannot comment. A qualified statistician is required.

Are all the source data underlying the results available to ensure full reproducibility?

Yes

Is the study design appropriate and is the work technically sound?

Yes

Are the conclusions drawn adequately supported by the results?

Yes

Are sufficient details of methods and analysis provided to allow replication by others?

Yes

Reviewer Expertise:

aquaculture

I confirm that I have read this submission and believe that I have an appropriate level of expertise to confirm that it is of an acceptable scientific standard.

# Peer review: Reviewer response for version 1

**DOI:** 10.5256/f1000research.77833.r121423

**Published:** 2022-02-16

**Authors:** Mohammad Tamrin Mohamad Lal

**Affiliations:** 1 Borneo Marine Research Institute, Universiti Malaysia Sabah, Kota Kinabalu, Malaysia

Overall, this manuscript is good. This paper can serve as a guideline for monitoring pollution in selected areas and can be used as a basis in other places. I recommend this paper for approval with further correction and clarification.

**Specific Comments**

 The title: I would suggest the title to be change to "Effect of environmental factors on blood counts of mosquito fish, *Gambusia affinis *caught at Brantas River watershed, Indonesia" because your result only focused on the blood counts and no other haemtological parameters (SOD, hemoglobin counts, respiratory burst, phagocytic index, etc.) 

 Abstract: Well written abstract. However, please remove "because it is" the sentence "This species was chosen because of its wide distribution along the Brantas River, and because it is very tolerant, adaptable, highly abundant, and easy to catch", as it is a repetition. 

**Introduction**

 The aim/objective of this study is not in line with the title and the conclusion. I would like to suggest the objective of this paper to be changed to "To evaluate the effect of physico-chemical water parameters on the blood counts of mosquito fish, *Gambusia affinis* in Brantas River".

**Methods**

 Some parts are not clear:

1. Put more details on the specific sampling time (morning, noon or evening).
2. What was the weather condition during the samplings?
3. How do you collect the water samples?
4. Is there any reason for not including the other water factors such as heavy metal concentration in this study?
5. Why don't you add male fish in this study? Is it that you won't be able to get the amount of blood needed? Since you did mention the fish is abundant in the area, therefore, the number of fish samples should not be a problem. Is there any reason for using small number of fish from each sampling location?
6. How do you do the blood sampling? Please put more detail on this (size of the needle, do you anaesthetize it? What method do you use to anaesthetize the fish? Do you refer to any specific method? Please cite it.
7. As for the blood analysis, is it a pooled samples from each sub-site? You should mention this.

**Result and Discussion**

 What is the meaning of (90) in this sentence .... the other region was higher (90)...?

 Delete "...discussed previously..."

 In the paragraph 6 of Hematology profile of *Gambusia affinis*, authors mentioned the fish sampled in this site experiencing health problem. However, in the earlier method for fish sampling, only healthy fish were sampled and in paragraph 8. This is contradicting sentences I found in this paper.

 Some references I found quite old. Probably the author can replace it with the newest reference.

Is the work clearly and accurately presented and does it cite the current literature?

Partly

If applicable, is the statistical analysis and its interpretation appropriate?

I cannot comment. A qualified statistician is required.

Are all the source data underlying the results available to ensure full reproducibility?

Yes

Is the study design appropriate and is the work technically sound?

Yes

Are the conclusions drawn adequately supported by the results?

Yes

Are sufficient details of methods and analysis provided to allow replication by others?

Yes

Reviewer Expertise:

aquaculture

I confirm that I have read this submission and believe that I have an appropriate level of expertise to confirm that it is of an acceptable scientific standard, however I have significant reservations, as outlined above.

# Response

**Published:** 2022-03-17

**Authors:** Asus Hertika

**Affiliations:** Lecturer at brawijaya university, Indonesia

Thank you for the comments from the reviewer of our article. In the following, we present clarifications as well as revisions to the manuscript that we have corrected

  

- Title: In this study, the haematology parameters were only measured on female fish, not all sexes. This must be clear.

*At first, we have tried to take blood sample from male fish. However, due to its small size, we had difficulties to take the blood samples. Therefore, in this research we took blood sample from the female fish in which its size was 3-4 times bigger than the male and the blood sample was easier to be taken. Furthermore, we using small number of fish from each sampling site because of several reasons. First, at each sampling site was consisted of 3 sub-sites and we consider it was representative. Second, it was due to research ethics code.*

- Abstract: Delete the words "and because it is" 

*Done*

 Methods - Things are not entirely clear.

  

- What is the sampling time? Is it the rainy or dry seasons and in the morning, afternoon, evening, and/or night for water quality and/or specimens?

*The sampling was done during noon and rainy season.*  

- How many times to take? For each sampling, how many times is sampling done as a replication for data validity, for both water quality and specimens.

*Sampling was replicated 3 times, every two weeks. AS each sampling site, we put samples from 3 sub-site. *

- In the current study, these months include the rainy season, how about during the dry season? Data is needed here too.

*We did not performed research during dry season. We plan for having sampling during dry season in the future.*

- What about COD and other water qualities, including hormones (EDC)? These should be included too.

*We did not measure these parameters. Thank you for the suggestion and we will consider these parameters for our nest research.  *

- How about nitrate/nitrite and heavy metals? Do these have no effect?

Jawab: *Nitrite and nitrate were not measured in this research, instead we measured ammonia. As for heavy metal, actually we also measured Hg, Pb and Cd. However, these variables were not presented in this manuscript, because it is being a part of another article which submitted in different journal.*

- What parameters are used as good health indicators? It would be better if the phenotypic or morphology performance of the fish was observed too.

*To make sure the fish sampled were healthy, we observed their morphological features.*

- The only fish observed were female: was it not possible to describe the entire population of fish (in terms of sex)?

*At first, we have tried to take blood sample from male fish. However, due to its small size, we had difficulties to take the blood samples. Therefore, in this research we took blood sample from the female fish in which its size was 3-4 times bigger than the male and the blood sample was easier to be taken. Furthermore, we using small number of fish from each sampling site because of several reasons. First, at each sampling site was consisted of 3 sub-sites and we consider it was representative. Second, it was due to research ethics code.*

- Is anesthesia done and what method was used when taking blood samples? This must be clear.

*We did not anaesthetize the fish*

- What about the other blood components? There should be measured data too.

*We did not measure other blood components such Hb because the blood sample volume was not sufficient to perform this measurement.*

  

- What about haemoglobin? This parameter is very important due to change of environmental conditions.

*We did not measure other blood components such Hb because the blood sample volume was not sufficient to perform this measurement.*

  

- What about haematocytes or monocyte and thrombocytes?

*Thank you for the suggestion to measure those parameters. We will consider them for our future research.*

 Results and Discussion:

 Water quality factors in Brantas River:

 Table 2:

- ‘While Malang is joint lowest with Batu, the gap between them is significant, and it is in fact closer in temperature to the other regions: explain why it is included with Batu.

*We have tried to explain it in the manuscript. *

- '...(90)...' what is this? A unit?  à *Thank you for your correction.*

*We believe that it is a writing error. We have deleted it from our manuscript*

- 'DO values'....Check again the data as in Table 2, out of sync!

*We have made correction, and add Tulungagung too as the region with high DO.*

- While Mojokerto has the lowest DO levels, they are not significantly different from the other areas, please explain the significance further.

 
*According to our data analysis, DO values in Mojokerto was the lowest and significantly different compared to other sites. It is because the notation letter of Tukey test for Mojokerto was not shared to other regions. In addition, the water quality condition of Mojokerto was explained altogether with other variables at the end of this section, which suggesting the poor condition of water quality in this site.*

- 'Thus, the higher...' Correct the sentences.

*We have made the correction.*

- 'Therefore, the majority...' Why? What caused it? This should be explained.

W *e have added explanation for this in the manuscript.*

 'The level of ammonia toxicity is influenced by several factors, including fish species...' How do the processes or mechanisms of fish species affect ammonia toxicity? This is not made clear.

 W *e have added explanation for this in the manuscript.*

 'Fish exposed to high levels ...' The sentences should be mentioned in the haematology results section.

*We briefly described the effect of ammonia on fish which related to water quality discussion.*

 'The physico-chemical conditions of the Brantas River ...' This statement is not in accordance with the data obtained (Table 2) because it is based on data that ammonia and phenol in all locations relatively exceed the standard limits.

*We explained that Batu as the least polluted region,* *since the values of its water quality factors were better than the other sites. Meanwhile, Mojokerto as the most polluted because the water quality was the poorest. We did not imply that other region were not polluted, since we emphasized on the pollution comparison between sites.*

 Hematology profile of Gambusia affinis caught in the Brantas River

- '... 25 cells/1000 or 10 cells/1000...' What is this (1000) unit?

*It is indicate cells unit*

- 'Erythrocytes are blood components which...' What about haemoglobin? This component plays a role very important too.

*We did not measure other blood components such Hb because the blood sample volume was not sufficient to perform this measurement.*

 Relationship analysis of hematology properties and water quality

 Relate and contrast the findings here ('Temperature, BOD, ...') with existing data, e.g. that found in Table 2 and Figure 2 (a and b).

*The relationship between these table and figure has been explicitly represented by Figure 3-5.*

 'As a result, high levels of ammonia cause ...' Check again as the data in the current study, they appear out of sync.

*We have checked the data, and as shown by Figure 3 and Figure 4, the statement is supported by our observed data.*

  

- It would be better if the histopathological results were added in the study, including to know anoxia, as described.

*Thank you for the suggestion. We will try to apply it for our next research.*

 'white blood cells ...' change to "leucocytes"

*Done*

  

- 'Genotoxicity testing of the aquatic ...' The sentences are more accurately mentioned in the Introduction section.

*Thank you. We have added it in the manuscript.*

# Peer review: Reviewer response for version 1

**DOI:** 10.5256/f1000research.77833.r100540

**Published:** 2022-01-11

**Authors:** Akhmad Mukti

**Affiliations:** 1 Department of Aquaculture, Faculty of Fisheries and Marine, Universitas Airlangga, East Java, Indonesia

**Title:** In this study, the haematology parameters were only measured on female fish, not all sexes. This must be clear.

**Abstract:** Delete the words "and because it is"

**Methods **- Things are not entirely clear.

  

1. What is the sampling time? Is it the rainy or dry seasons and in the morning, afternoon, evening, and/or night for water quality and/or specimens?
2. How many times to take? For each sampling, how many times is sampling done as a replication for data validity, for both water quality and specimens.
3. In the current study, these months include the rainy season, how about during the dry season? Data is needed here too.
4. What about COD and other water qualities, including hormones (EDC)? These should be included too.
5. How about nitrate/nitrite and heavy metals? Do these have no effect?
6. What parameters are used as good health indicators? It would be better if the phenotypic or morphology performance of the fish was observed too.
7. The only fish observed were female: was it not possible to describe the entire population of fish (in terms of sex)?
8. Is anesthesia done and what method was used when taking blood samples? This must be clear.
9. What about the other blood components? There should be measured data too.
10. What about haemoglobin? This parameter is very important due to change of environmental conditions.
11. What about haematocytes or monocyte and thrombocytes?

 

**Results and Discussion:**

*Water quality factors in Brantas River:*

 Table 2:

- ‘While Malang is joint lowest with Batu, the gap between them is significant, and it is in fact closer in temperature to the other regions: explain why it is included with Batu.
-  '...(90)...' what is this? A unit? 
- 'DO values'....Check again the data as in Table 2, out of sync!
- While Mojokerto has the lowest DO levels, they are not significantly different from the other areas, please explain the significance further.

Furthermore:

1. 'Thus, the higher...' Correct the sentences.
2. 'Therefore, the majority...' Why? What caused it? This should be explained.
3. 'The level of ammonia toxicity is influenced by several factors, including fish species...' How do the processes or mechanisms of fish species affect ammonia toxicity? This is not made clear.
4. 'Fish exposed to high levels ...' The sentences should be mentioned in the haematology results section.
5. 'The physico-chemical conditions of the Brantas River ...' This statement is not in accordance with the data obtained (Table 2) because it is based on data that ammonia and phenol in all locations relatively exceed the standard limits.

*Hematology profile of* Gambusia affinis *caught in the Brantas River*

1. '... 25 cells/1000 or 10 cells/1000...' What is this (1000) unit?
2. 'Erythrocytes are blood components which...' What about haemoglobin? This component plays a role very important too.

*Relationship analysis of hematology properties and water quality*

1. Relate and contrast the findings here ('Temperature, BOD, ...') with existing data, e.g. that found in Table 2 and Figure 2 (a and b). 
2. 'As a result, high levels of ammonia cause ...' Check again as the data in the current study, ****they appear out of sync.
3. It would be better if the histopathological results were added in the study, including to know anoxia, as described.
4. 'white blood cells ...' change to "leucocytes".
5. 'Genotoxicity testing of the aquatic ...' The sentences are more accurately mentioned in the Introduction section.

Please also see annotated manuscript for further comments, as well as further context for points raised here.

Is the work clearly and accurately presented and does it cite the current literature?

Yes

If applicable, is the statistical analysis and its interpretation appropriate?

Yes

Are all the source data underlying the results available to ensure full reproducibility?

Yes

Is the study design appropriate and is the work technically sound?

Yes

Are the conclusions drawn adequately supported by the results?

Yes

Are sufficient details of methods and analysis provided to allow replication by others?

Partly

Reviewer Expertise:

Fish genetics, fish reproduction, an endocrine disorder, genotoxic, karyology, aquaculture biotechnology

I confirm that I have read this submission and believe that I have an appropriate level of expertise to confirm that it is of an acceptable scientific standard, however I have significant reservations, as outlined above.

# Response

**Published:** 2022-03-17

**Authors:** Asus Hertika

**Affiliations:** Lecturer at brawijaya university, Indonesia

Thank you for the comments from the reviewer of our article. In the following, we present clarifications as well as revisions to the manuscript that we have corrected

 Specific Comments

  

- The title: I would suggest the title to be change to "Effect of environmental factors on blood counts of mosquito fish, Gambusia affinis caught at Brantas River watershed, Indonesia" because your result only focused on the blood counts and no other haemtological parameters (SOD, hemoglobin counts, respiratory burst, phagocytic index, etc.)

*The title has changed accordingly*

  

- Abstract: Well written abstract. However, please remove "because it is" the sentence "This species was chosen because of its wide distribution along the Brantas River, and because it is very tolerant, adaptable, highly abundant, and easy to catch", as it is a repetition.

W *e have made change to the abstract. Thank you for the correction.*

 Introduction

  

- The aim/objective of this study is not in line with the title and the conclusion. I would like to suggest the objective of this paper to be changed to "To evaluate the effect of physico-chemical water parameters on the blood counts of mosquito fish, Gambusia affinis in Brantas River".

*Thank you for the suggestion. We have changed the study purpose as suggested*

 Methods

 Some parts are not clear:

- Put more details on the specific sampling time (morning, noon or evening).

*The sampling was conducted at noon*

- What was the weather condition during the samplings?

*The weather was rainy*

- How do you collect the water samples?

*Temperature, pH, and DO were measured in-situ. Meanwhile, water samples to measure the rest parameters were taken as much as 1 liter, then put to the cold box. Afterward, the samples were analyzed at laboratory.*

  

- Is there any reason for not including the other water factors such as heavy metal concentration in this study?

*In this research, we also measured heavy metals such as Hg, Cd, and Pb. However, the results were not presented in this manuscript. It is because they are part of another article which is being submitted to other journal. *

- Why don't you add male fish in this study? Is it that you won't be able to get the amount of blood needed? Since you did mention the fish is abundant in the area, therefore, the number of fish samples should not be a problem. Is there any reason for using small number of fish from each sampling location? à *we chose only female fish because it has larger body size than male. *

*At first, we have tried to take blood sample from male fish. However, due to its small size, we had difficulties to take the blood samples. Therefore, in this research we took blood sample from the female fish in which its size was 3-4 times bigger than the male and the blood sample was easier to be taken. Furthermore, we using small number of fish from each sampling site because of several reasons. First, at each sampling site was consisted of 3 sub-sites and we consider it was representative. Second, it was due to research ethics code. *

- How do you do the blood sampling? Please put more detail on this (size of the needle, do you anaesthetize it? What method do you use to anaesthetize the fish? Do you refer to any specific method? Please cite it.

*The blood sampling method is explained in Fish Blood Sample Collection and Hematology Study. In addition, we did not anaesthetize the fish.*

  

- As for the blood analysis, is it a pooled samples from each sub-site? You should mention this.

*Yes, it is true that the samples were pooled from each sub-site. We have added this to the manuscript*

 Result and Discussion

  

- What is the meaning of (90) in this sentence .... the other region was higher (90)...?

*Thank you for your correction. We believe that it is a writing error. We have deleted it from our manuscript*

 Delete "...discussed previously..."

 D *one*

  

- In the paragraph 6 of Hematology profile of Gambusia affinis, authors mentioned the fish sampled in this site experiencing health problem. However, in the earlier method for fish sampling, only healthy fish were sampled and in paragraph 8. This is contradicting sentences I found in this paper. 

 
*As mentioned in our methodology, we chose health fish in accordance to its appearance. However, since the leucocyte and erythrocyte count of the sampled fish were quite low, then it might indicate the presence of fish’ health problem internally that probably did not visible appearance wise.*

- Some references I found quite old. Probably the author can replace it with the newest reference.

W *e have replaced the reference before 2000 with the updated ones.*
